# Analysis of Replication, Cell Division-Mediated Spread, and HBV Envelope Protein-Dependent Pseudotyping of Three Mammalian Delta-like Agents

**DOI:** 10.3390/v16060859

**Published:** 2024-05-28

**Authors:** Gnimah Eva Gnouamozi, Zhenfeng Zhang, Vibhu Prasad, Chris Lauber, Stefan Seitz, Stephan Urban

**Affiliations:** 1 Department of Infectious Diseases, Molecular Virology, University Hospital Heidelberg, 69120 Heidelberg, Germany; gnimaheva.gnouamozi@med.uni-heidelberg.de (G.E.G.); zhangzf@sustech.edu.cn (Z.Z.); vibhu.prasad@med.uni-heidelberg.de (V.P.); 2 School of Public Health and Emergency Management, Southern University of Science and Technology, Shenzhen 518055, China; 3 Institute for Experimental Virology, TWINCORE Centre for Experimental and Clinical Infection Research, a Joint Venture between the Hannover Medical School (MHH) and the Helmholtz Centre for Infection Research (HZI), 30625 Hannover, Germany; chris.lauber@twincore.de; 4 German Center for Infection Research (DZIF), Hannover Partner Site, 38124 Hannover, Germany; 5 Cluster of Excellence 2155 RESIST, 30625 Hannover, Germany; 6 German Center for Infection Research (DZIF), Heidelberg Partner Site, 69120 Heidelberg, Germany; s.seitz@dkfz-heidelberg.de; 7 German Cancer Research Center (DKFZ), Division of Virus-Associated Carcinogenesis, 69120 Heidelberg, Germany

**Keywords:** hepatitis delta virus, HDV, HDV evolution, mammalian delta-like agents, cell division-mediated spread

## Abstract

The human hepatitis delta virus (HDV) is a satellite RNA virus that depends on hepatitis B virus (HBV) surface proteins (HBsAg) to assemble into infectious virions targeting the same organ (liver) as HBV. Until recently, the evolutionary origin of HDV remained largely unknown. The application of bioinformatics on whole sequence databases lead to discoveries of HDV-like agents (DLA) and shed light on HDV’s evolution, expanding our understanding of HDV biology. DLA were identified in heterogeneous groups of vertebrates and invertebrates, highlighting that the evolution of HDV, represented by eight distinct genotypes, is broader and more complex than previously foreseen. In this study, we focused on the characterization of three mammalian DLA discovered in woodchuck (*Marmota monax*), white-tailed deer (*Odocoileus virginianus*), and lesser dog-like bat (*Peropteryx macrotis*) in terms of replication, cell-type permissiveness, and spreading pathways. We generated replication-competent constructs expressing 1.1-fold over-length antigenomic RNA of each DLA. Replication was initiated by transfecting the cDNAs into human (HuH7, HeLa, HEK293T, A549) and non-human (Vero E6, CHO, PaKi, LMH) cell lines. Upon transfection and replication establishment, none of the DLA expressed a large delta antigen. A cell division-mediated viral amplification assay demonstrated the capability of non-human DLA to replicate and propagate in hepatic and non-hepatic tissues, without the requirement of envelope proteins from a helper virus. Remarkably L-HDAg but not S-HDAg from HDV can artificially mediate envelopment of WoDV and DeDV ribonucleoproteins (RNPs) by HBsAg to form infectious particles, as demonstrated by co-transfection of HuH7 cells with the respective DLA expression constructs and a plasmid encoding HBV envelope proteins. These chimeric viruses are sensitive to HDV entry inhibitors and allow synchronized infections for comparative replication studies. Our results provide a more detailed understanding of the molecular biology, evolution, and virus–host interaction of this unique group of animal viroid-like agents in relation to HDV.

## 1. Introduction

The human hepatitis delta virus (HDV) is the human virus with the smallest RNA genome. As a satellite virus, HDV needs the presence of the glycoproteins of its helper, hepatitis B virus (HBV), to assemble into viral particles able to egress from infected hepatocytes. The HBV envelope proteins also mediate specific entry via Heparan sulfate proteoglycan (HSPG) and Na+-taurocholate co-transporting polypeptide (NTCP) into hepatocytes. Since its discovery in 1977 [1], the association between HDV and HBV is considered exclusive, confining the HDV tropism uniquely to hepatocytes. The discovery of HDV-like agents (DLA) in vertebrate and invertebrate species overthrew the long-lasting notions about HDV as the only representative of the *deltavirus* genus. Notably, the replication of these novel delta agents was detected in other organs than the liver without the presence of respective hepadnaviruses, strengthening the notion that the unique hepatotropism of HDV is mediated by the HBV envelope. Wille et al. reported HDV-like sequences detected in a pool of oropharyngeal and cloacal samples of diverse species of duck (*Anas gracilis*, *Anas castanea*, and *Anas superciliosa*) [2]. Snake delta-like RNA was found in various tissues of two snake species (*Boa constrictor* and *Liasis fuscus*) such as the liver, spleen, kidney, lung, and brain, indicating a broad cellular tropism [3]. Moreover, in 2019, further DLA were identified in fish, amphibians, and invertebrates [4]. Mammalian HDV-like sequences were also discovered in liver, cardiac, lung, intestinal, and kidney samples from a spiny rat (*Proechimys semispinosus*), liver samples from woodchuck (*Marmota monax*), pedicle of a white-tailed deer (*Odocoileus virginianus*), and liver of two species of bat (*Peropteryx macrotis* and *Desmodus rotundus*) [5,6,7]. The ORFs of the respective delta antigens share significant homology with the human small delta antigen (S-HDAg), indicating that HDV and all DLA are evolutionarily linked (Figure S1).

In addition to the extracellular HBV envelope protein-mediated spreading pathway, HDV can also propagate through cell division [8,9]. While the de novo infection via NTCP binding can be blocked by the entry inhibitor Bulevirtide (BLV), cell division-mediated intrahepatic spread (CDMS) is resistant to BLV but sensitive to interferon (IFN) [9]. The ISGs responsible for the inhibition of cell division-mediated RNA amplification are only partially understood and characterized. Nevertheless, this envelope-protein-independent way of propagation might be also important for DLA, in the absence of a helper virus.

For packaging by HBsAg, HDV relies on the editing of its antigenome by the cellular enzyme Adenosine Deaminase Acting on RNA-1 (ADAR1), which leads to the mutation of a stop codon (UAG) at the C-terminus of its S-HDAg coding sequence to a tryptophan codon (UGG). This results in the elongation of S-HDAg by 19 or 20 amino acids (genotype dependent) and, in a timely regulated manner, to the expression of the large delta antigen (L-HDAg) [10]. The extension contains a prenylation motif (CXXQ) that, when becoming farnesylated at the Cys-211 residue, is crucial for the interaction between the L-HDAg, as part of the ribonucleoprotein complex, and HBsAg [11,12,13]. In silico sequence analyses predict that all DLA described so far do not encode a large delta antigen containing a prenylation motif. This supports the hypothesis that DLA do not depend on hepadnaviral envelope proteins containing a prenylation recognition sequence within their cytosolic loop but evolved alternative mode(s) of propagation, including adaptation to possible other viral envelopes or even propagate without helper virus-encoded envelope proteins. Regarding the first hypothesis, in the case of the avian delta agent, sequences of influenza A virus genome have been identified in the same swabs [2]; similarly, it was demonstrated that replication and extracellular spread of the snake delta agent occurs in the presence of both reptarenaviruses and hartmaniviruses [3,14,15]. Furthermore, the mammalian delta agents were found to be coinfecting with poxviruses, hepaciviruses, and retroviruses, with no trace of hepadnaviral co-infection [5,7]. Whether these viruses bear a true helper function for the respective DLA remains to be investigated. Regarding the second hypothesis, the possibility of an extracellular spread independent from a helper virus should be considered. Indeed, considering the similarities between DLA and viroids infecting plants, which propagate in absence of helper virus via mechanical transmission of RNA molecules, it would be tempting to speculate on a similar spreading pathway for DLA too. In this work, we characterized a panel of DLA with respect to cellular permissiveness, replication competence, propagation pathways, and putative virion formation.

## 2. Materials and Methods

### 2.1. HDV and HDV-like Agents Plasmids

Plasmid pJC126 harboring 1.1 copy of the HDV-genotype 1 antigenome was kindly provided by Prof. Dr. John Taylor. Plasmids pcDNA3.1/Zeo (+) containing 1.1 copies of the antigenome of HDV-like agents were generated by the insertion of a synthetic 1.1-HDV antisense sequence into plasmid pcDNA3.1 Zeo (+). Further details are provided in Table S1.

### 2.2. Cell Division-Mediated Amplification Assay and Cluster Analysis

Cell lines were transfected with plasmids encoding a 1.1-fold antigenome of HDV or respective DLA. At day 5 post-transfection (passage 0 = P0), cells were split at different dilution factors (1:20, or 1:100) to allow clonal expansion. Before every split, cells were fixed in parallel, and delta antigen expression was visualized by immunofluorescence (IF) analysis using the FD3A7 monoclonal antibody against S-HDAg. The cluster quantification analysis was performed using Cell Profiler software 4.2.1. For identification of fluorescent focusing units (FFU), a software package implementing source executed Cellprofiler 4.2.1 with Cellpose 2 was used, as described before [16]. In brief, the cells were segmented as objects using DAPI signal and objects were merged into a single FFU if they are within an arbitrary distance of 80 pixels to each other. This pixel number was determined manually based on a test image with well-separated FFUs. Based on these merged objects as FFUs, intensity and area were measured. To account for both the area and intensity of the clusters (FFUs), a combined measurement of area*intensity was used.

### 2.3. HDV, WoDV/HBsAg and DeDV/HBsAg Pseudoparticles Production

HuH7 cells were seeded at a density of 3 × 10^6^ cells per 10 cm dish. The following day, cells were transfected with 2.5 µg of delta agent plasmids and 2.5 µg of plasmid expressing HBV envelope proteins (pT7HB2.7) using LT1 Transfection reagent (product No. MIR 2305). Three days post-transfection (p.t.), the producer cells were transfected again with a plasmid encoding for L-HDAg. The supernatant was collected on days 7, 10, and 13 post-transfection, and viral particles were precipitated using Polyethylene Glycol 8000 (PEG8000). To precipitate the viral particles, PEG8000 (40% stock solution) was added to the collected supernatant to a final concentration of 10%. The solution was incubated overnight at 4 °C, then centrifuged for 1 h at 10,000× *g*. The precipitate was recovered in 100 µL of 10% FCS/PBS and incubated with shaking at 4 °C overnight to allow complete resuspension of viral particles. After resuspension, the viral stock was centrifuged twice at 3000× *g* for 10 min at room temperature to eliminate the insoluble portion. The virus preparation was aliquoted and stored at −20 °C or −80 °C for long-term storage.

Further details regarding material and methods are described in Supplementary Materials.

## 3. Results

### 3.1. Establishment and Characterization of HDV-like Agents Infectious Clones

**Figure 1:**
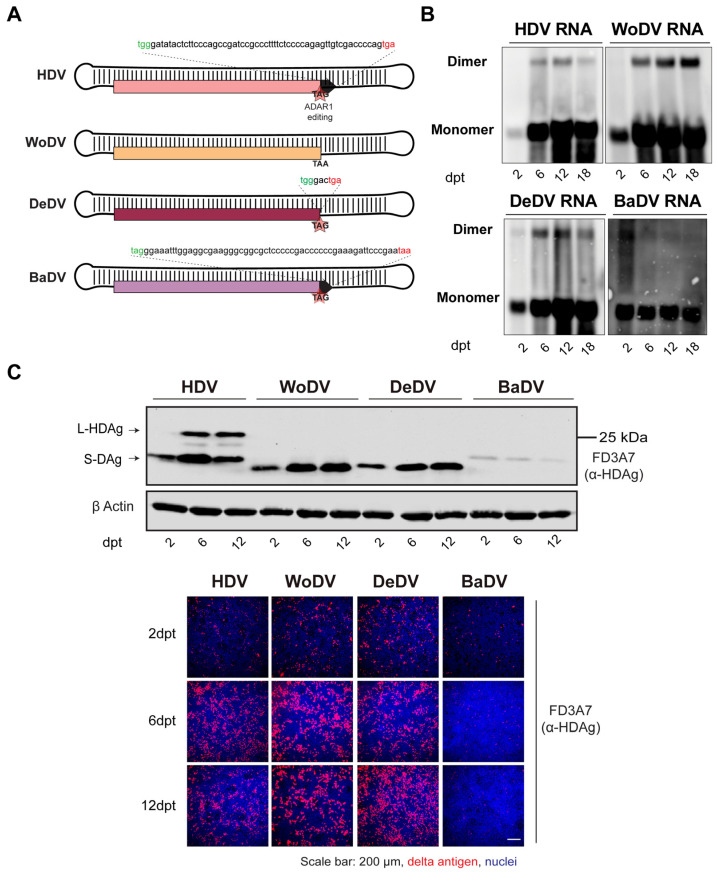
Characterization of antigenomic cDNA clones of the mammalian DLA identified in woodchuck (WoDV), deer (DeDV), and bat (BaDV). (**A**) Schematic representation of the putative large delta antigen generated by ADAR1 editing of the DLA genomes. (**B**) HuH7 cells were transfected with pcDNA3.1 plasmid harboring a 1.1-fold DLA antigenome and harvested at day 2-, 6-, 12-, and 18-p.t. for viral RNA detection by Northern blots using genome-specific DIG-labeled probes. (**C**) Delta antigen (DAg) expression was visualized via Western blot analysis (**upper panel**) or via immunofluorescence staining (**lower panel**) using FD3A7 anti-S-HDAg antibody at d2-, 6-, 12 p.t.

To assess the replication competence and the ability to produce a large delta antigen (Figure 1A), HuH7 cells were transfected with pcDNA3.1 plasmid harboring the 1.1-fold DLA antigenome (Table S1 and Figure S2).

To provide a comparison between HDV and HDV-like agents, cells were transfected with a pcDNA3.1/Zeo (+) plasmid harboring 1.1mer antigenomic sequence of HDV (pJC126), used as replication competent control, while an HDV genotype 5 replication defective clone (HDV defect) was used as negative control to define lack of replication capacity (Figure S3) [17].

Intracellular viral RNA levels of the woodchuck (WoDV) and deer (DeDV) delta agents, visualized by Northern Blot (NB), increased in transfected HuH7 starting from day 2 post-transfection (p.t.) until day 18 p.t. (Figure 1B). Western blot (WB) analysis (Figure 1C upper panel) showed a small delta antigen (DAg) expression for WoDV and DeDV, increasing from d6 p.t. to d12 p.t. The bat delta agent (BaDV) antigen expression was found to be weaker compared to the other mammal counterparts, and no significant increase from d2 p.t. to d6 p.t. was observed, indicating inefficient replication of BaDV in HuH7 cells. Importantly, no large delta antigen (L-Ag) expression was detected for WoDV, DeDV, and BaDV, confirming the in silico prediction data (Figure 1C and Figure S1).

### 3.2. WoDV and DeDV RNA Can Amplify Efficiently via Cell Division

**Figure 2:**
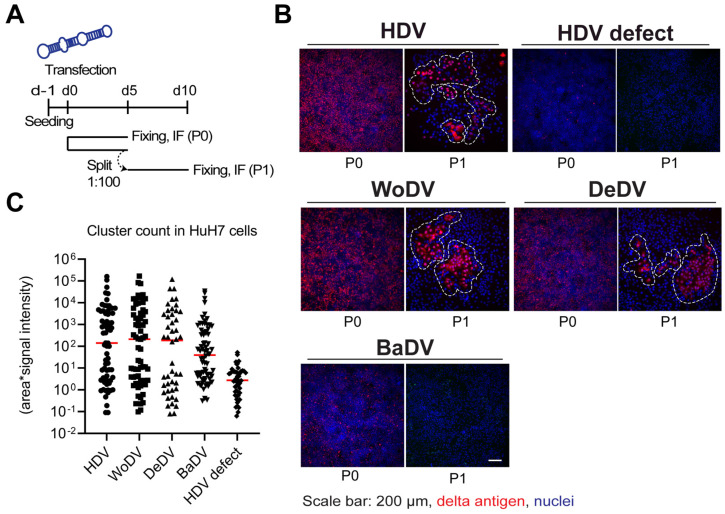
WoDV, DeDV, and BaDV cell division-mediated spread in HuH7 cells. (**A**) Cells were transfected with DLA constructs and split (1:100-dilution) at d5 p.t. (passage 0 = P0). Cells were fixed on day 5 p.t. or day 5 post passage (P1). (**B**) Delta antigen expression was visualized by immunofluorescence using FD3A7 antibody. (**C**) Delta antigen-positive cells were then quantified using Cell Profiler software 4.2.1. Quantification was carried out taking into consideration cluster size and mean signal intensity.

HDV can spread and persist intracellularly in daughter cells after cell division [7,8]. To investigate if this holds true for the mammalian delta agents investigated here, we performed a cell division-mediated viral amplification assay. HuH7 cells were transfected with 1.1-fold antigenome constructs and split every 5 days by 1:100-fold dilution. Before every split, one control well of transfected cells was fixed and DAg-positive cells were analyzed by immunofluorescence (Figure 2A). Since neither envelope proteins of HBV nor NTCP are present in this system, virion-mediated spreading of RNA can be excluded. Furthermore, since HuH7 cell lines are deficient in interferon (IFN) production, cell division-mediated spread is not restricted by IFN.

In HuH7 cells, clusters of delta antigen positive cells were detected for WoDV and DeDV from passage 1 (P1) (Figure 2B,C) to passage 3 (P3, comparable to the human HDV. Clusters of positive cells were also detected for BaDV but only when a lower dilution factor during splitting was applied, indicating a lower replication rate for BaDV in HuH7 cells. To distinguish between CDMS and extracellular cell-to-cell spread possibly mediated by cellular vesicles, we generated a co-culture system based on fluorescent protein expression. After 12 days of co-culture, non-transfected cells did not become delta antigen positive, indicating the lack of autonomous virus transmission between adjacent cells (Figure S4).

### 3.3. Delta-like Agents Show a Wide Host Range for Replication

**Figure 3:**
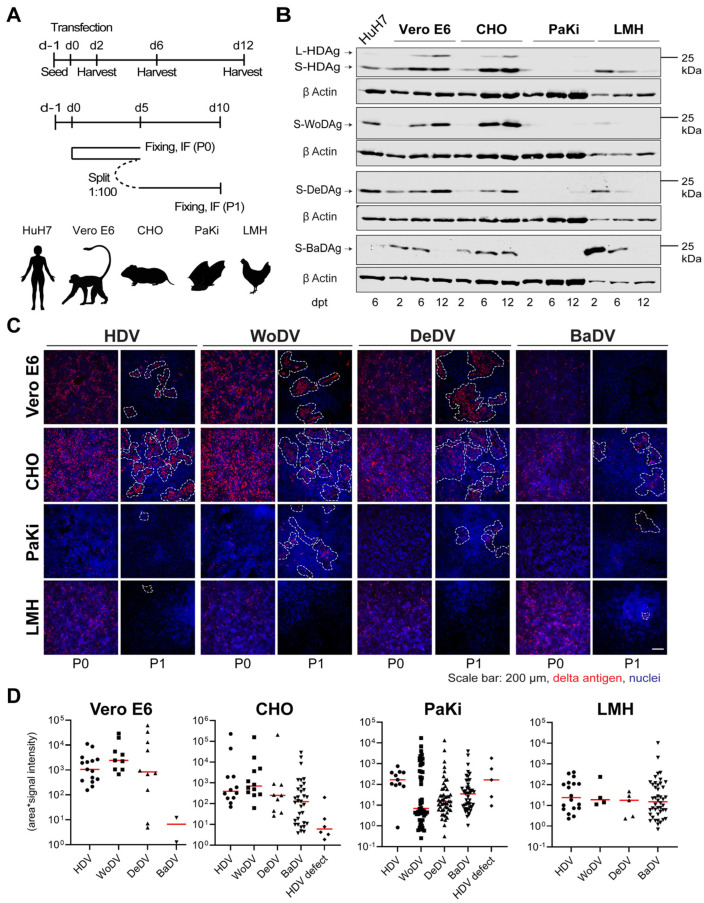
HDV and DLA viral antigen expression and cell division-mediated spread in different animal cell lines. (**A**) Vero E6, CHO, PaKi, and LMH cells were transfected with plasmids encoding the 1.1 antigenomic cDNA of HDV, WoDV, DeDV, and BaDV (**upper panel**, related to (**B**)). In parallel, cells were transfected and split (1:100-dilution) at d5 p.t. (passage 0 = P0) (**lower panel**, related to (**C**,**D**)). (**B**) Antigen expression was analyzed at d2, d6, and d12 p.t. following total cell lysis by WB. (**C**) At d5 p.t. or d5 post passage (P1), cells were fixed, and delta antigen expression was visualized by IF analysis using FD3A7 antibody. (**D**) Clusters of delta antigen-positive cells were quantified in terms of size and signal intensity using Cell Profiler software 4.2.1.

The weak replication of BaDV in HuH7 cells and its phylogenetic distance to HDV prompted us to investigate the host range of HDV and DLA. We therefore compared replication kinetics and CDMS of BaDV and the other mammalian delta-like agents in 4 different animal cells (Figure 3A): Vero E6 (African Green Monkey—Kidney), CHO (Chinese hamster—Ovary), PaKi (Black Flying Fox Bat—Kidney), and LMH (Chicken—Liver). The expression of HDV S- and L-HDAg was observed in VeroE6 and CHO cells, while the signal was weak in Paki and absent in LMH cells (Figure 3B). HDV, WoDV, and DeDV showed high replication in VeroE6 and CHO cells, but no L-Ag expression was observed for WoDV or DeDV, confirming results in HuH7 cells. BaDV Ag expression increased over time only in CHO cells but was still at a lower level when compared to other mammalian DLA. No expression increase in BaDAg was found in Paki cells, suggesting the need to validate the replication of this agent in a broader selection of bat-derived cell lines.

Vero E6 and CHO cells exhibited efficient CDMS of HDV, WoDV, and DeDV (Figure 3C,D), consistent with the pattern observed for Ag expression kinetics. Lower clustering was observed in PaKi and LMH cells for HDV, WoDV, and DeDV, as indicated by the smallest cluster size and weaker signal intensity. Interestingly, no cluster was formed by BaDV in the bat-derived cell line (Figure 3C,D).

### 3.4. Cell Division-Mediated Spread of DLA in Non-Hepatic Cell Lines

**Figure 4:**
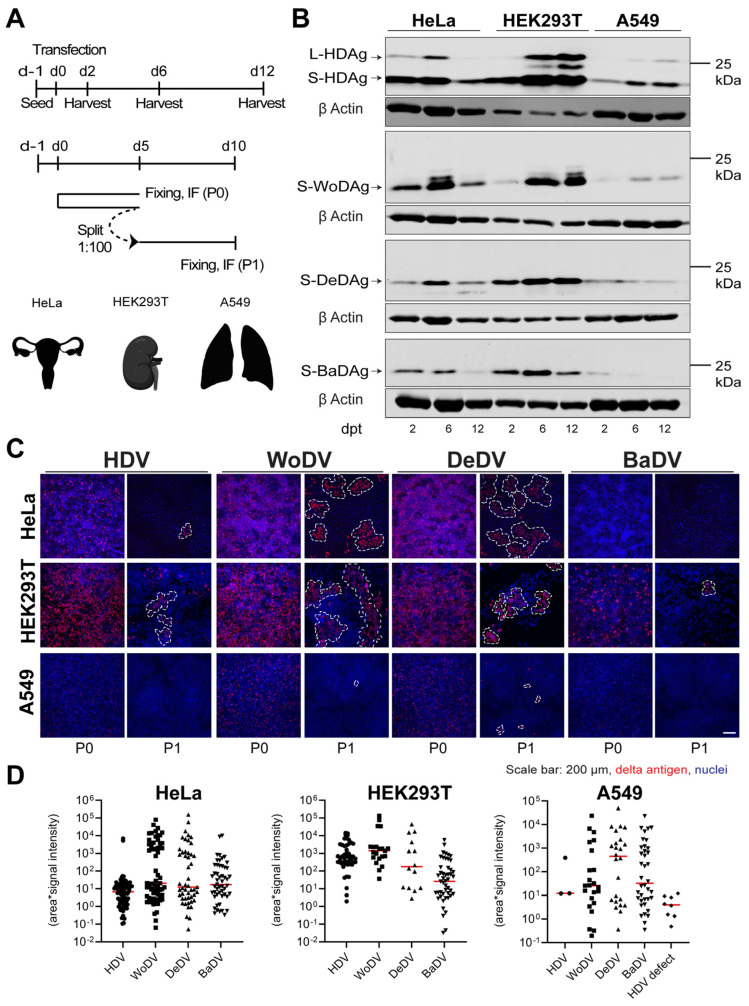
WoDV, DeDV, and BaDV viral antigen expression and cell division-mediated spread in non-hepatic human cells. (**A**) HeLa, HEK293T, and A549 cells were transfected with DLA 1.1mer antigenome constructs (**upper panel**, related to (**B**)). In parallel, cells were transfected and split (dilution factor 1:100) at day 5 p.t. (passage 0 = P0) (**lower panel**, related to (**C**,**D**)). (**B**) Delta antigen expression was analyzed at d2, d6, and d12 p.t. (**C**) At day 5 p.t. or day 5 post-passage (P1), cells were fixed and stained for delta antigen visualization by IF using FD3A7 antibody. (**D**) Cluster of delta antigen-positive cells were quantified using Cell Profiler software 4.2.1.

Since sequences of DLA have been found in non-liver tissues, it is reasonable that they do not preferentially replicate in the liver of their respective hosts. To investigate cellular permissiveness in cells different from hepatic origin, we transfected three human non-hepatic cell lines (HeLa- Cervix, HEK293T-Kidney, and A549-Lung) with the cDNAs of DLA (Figure 4A) and assessed viral antigen expression at day 2, 6, and 12 p.t. (Figure 4A-upper scheme and B). To evaluate CDMS, we performed, in parallel, a cell division-mediated viral amplification assay (Figure 4A, lower scheme). Shortly before splitting a control well, transfected cells were fixed in parallel and immunoassayed for delta antigen expression.

In HeLa and HEK293T cells, WoDV and DeDV show strong enrichment of delta antigens, indicating the immediate onset of amplification of RNA (Figure 4B). Also, CDMS was observed and was even more efficient when compared to HDV (Figure 4C,D). Notably, for neither HDV nor for the DLA was the formation of delta antigen-positive clusters after CDMS observed in A549 cells (Figure 4C,D). Possible explanations could be the lack of tissue-specific host factors or the presence of an active restriction factor not allowing an efficient replication in lung-derived cell lines. Moreover, an important limiting factor might be the role of the innate immune system in the establishment of replication, considering the innate immunity competency of A549 cells.

### 3.5. WoDV and DeDV Can Exploit HDV L-HDAg for Envelopment and NTCP-Receptor-Mediated Entry into Hepatocytes

**Figure 5:**
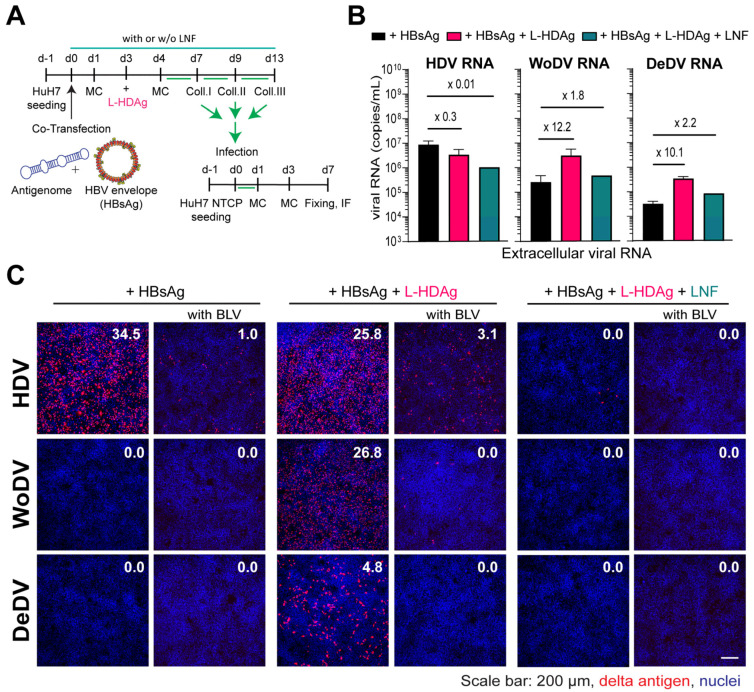
L-HDAg complementation and packaging by HBV envelope proteins. (**A**) HuH7 cells were co-transfected with DLA 1.1-mer antigenome constructs and pT7-HB2.7 plasmid with or without L-HDAg trans-complementation. (**B**) Supernatant from transfection was collected and, after PEG precipitation, the viral load was measured via RT-qPCR. (**C**) PEG precipitated virus was used to infect HuH7NTCP cells, and after 7 days, cells were fixed and stained for delta Ag visualization (FD3A7 anti-S-HDAg and Alexa Fluor 546-labeled anti-rabbit antibody). The first column represents infection performed with virus produced without L-HDAg complementation that was also performed in parallel with Bulevirtide (BLV) treatment (**second column**). The third and fourth columns depict infection with L-HDAg complemented virus with or without BLV treatment, respectively. The fifth and sixth columns depict infection with L-HDAg complemented virus and Lonafarnib (LNF) treatment (1 µM) to prevent farnesylation, with or without BLV treatment, respectively. Delta antigen-positive cells were quantified using the Ilastik program, as indicated by the percentage of infected cells.

To elucidate whether DLA RNPs can be complemented and functionally interact with the prenylated form of the HDV-derived L-HDAg and thereby gain the possibility to become enveloped by the envelope glycoproteins of HBV, we sought to pseudo-type the RNPs of DLA and produce infectious particles for infectivity assays. To that aim, we transfected HuH7 with antigenome-expressing plasmids together with an HBsAg plasmid. Three days later, HuH7 cells were additionally transfected with a plasmid encoding for the human L-HDAg (Figure 5A). To assess whether envelopment was specifically mediated by the interaction between HBsAg and exogenous farnesylated L-HDAg, virus production was also performed under Lonafarnib (LFN) treatment, which inhibits farnesylation and consequently viral particle formation.

L-HDAg trans-complementation for WoDV and DeDV DLA led to an increase in viral RNA release into the supernatant, which was reduced under LFN treatment (Figure 5B). To test the infectivity of pseudo-typed viral particles, comparable amounts of PEG precipitated virus (in µL) were used for infecting HuH7NTCP cells. While co-transfection of HDV with HBsAg resulted in the secretion of infectious particles, the packaging was not observed for WoDV and DeDV (Figure 5C, left panel), confirming previous findings from Iwamoto et al., where no HBsAg envelopment was seen for WoDV and DeDV [6]. Remarkably, the trans-complementation of L-HDAg yielded a high infection rate for WoDV and DeDV (Figure 5C, middle panel). The entry inhibitor BLV proficiently blocked the infection, indicating an authentic NTCP-dependent virus entry. No infection events could be observed when producing cells were treated with LFN (Figure 5C, right panel).

In summary, HDV-like agents were able to establish replication in human (HuH7, HeLa, HEK293T) and non-human (VeroE6, CHO) cell lines. HDV-like agents like HDV efficiently spread via cell division, underpinning the general importance of this spreading pathway, not only in the context of HDV. However, among all tested cell lines, PaKi, LMH, and A549 show little permissiveness for viral replication for reasons that still have to be clarified.

Remarkably, L-HDAg complementation allowed the packaging of WoDV and DeDV RNP by HBsAg, indicating that only the integration of a small extension to the S-DAg encoding a prenylation signal allowed the virus to adapt to hepadnaviruses. The viral pseudo-particles produced established infection in HuH7NTCP cells via NTCP receptor binding.

## 4. Discussion

DLA from diverse animal species have been identified in recent years. All of them share common but also distinct genome characteristics when compared to human HDV. The most important difference is the deficiency for expression of a farnesylated large delta antigen. When farnesylated by cellular farnesyltransferase, L-HDAg from HDV negatively regulates HDV replication [18,19,20] and enables the HDV RNP to interact with the self-assembly competent envelope proteins of HBV to form HDV particles [11,12,13].

In our study, we generated cDNA constructs of three mammalian DLA that were capable of establishing replication in different human- and non-human-derived cell lines. Efficacy of replication and kinetics of RNA amplification differed markedly. Following transfection of HuH7 cells with cDNAs of WoDV, DeDV, and BaDV, only the respective small delta antigens (Figure 1C) were detectable, implying that RNA modifications (e.g., ADAR editing) that lead to extension of the ORFs cannot be achieved. BaDV delta antigen expression was lower when compared to WoDV and DeDV antigens, suggesting either species-specific differences in their replication rate or potential differences in promoter regions within the BaDV genome when compared to the other mammalian counterparts. Whether these differences are due to specific host factor dependencies shall be elucidated in further studies.

Application of a recently developed cell division-mediated RNA amplification assay allowed us to demonstrate the capability of WoDV, DeDV, and BaDV, in comparison to HDV, to amplify their genomes via clonal expansion of single infected/transfected cells (Figure 2), indicating that this spreading pathway is not restricted to HDV but has evolutionarily evolved within DLA before adaptation to HBV envelopment [8,9].

HDV replication proceeds via a double rolling circle mechanism, a strategy shared with plant-infecting viroids. The common small RNA genome size and replication mechanism contributed to the hypothesis that sees HDV having originated from viroids with subsequent acquisition of the protein HDAg [21,22,23]. Considering these similarities, HDV and DLA might be able to spread and amplify without the requirement of an envelope, via the induction of cell–cell plasma-membrane fusion, promoting a direct movement of RNPs among adjacent cells. However, we did not observe any viral spread from infected to neighboring cells in our co-culture system (Figure S4), confirming the observation reported in an earlier study where only HDV was investigated [8].

The discovery of HDV-like agents in a broad range of species highlights the possibility of host-shifting [5]. Accordingly, our findings show that these agents replicate in diverse animal-derived cell lines like VeroE6 (monkey) and CHO (hamster). Moreover, in the same cell lines, an efficient CDMS for WoDV and DeDV supports this assumption. However, the highly reduced replication in galliform (LMH)- and chiroptera (PaKi)-derived cell lines (Figure 3) possibly hints at a preference for replication in mammalian-derived cell lines. A broad host cell range was also shown by Khalfi et al. in the context of the rodentDV (RoDV) replication when compared to the snakeDV (SDeV) replication [24].

Surprisingly, BaDV could not efficiently replicate even in a bat cell line. Whether the cell line lacks essential factors for BaDV replication and the role of environmental temperature changes in replication fitness will be further investigated.

While 1.1-mer DNA clones are useful for initiating and monitoring replication establishment, there are significant challenges in comparing replication efficiency between different cell lines and clones. Firstly, the expression and functionality of the viral delta antigen can vary between cell types. Secondly, the efficiency of the poly-A site during mRNA synthesis may vary between different DLA clones, potentially affecting replication outcomes. Finally, one should consider potential differences between a single clone and the actual viral agent, such as potential sequence variations that may affect replication.

Metagenome analysis led to the detection of genetic fragments of these novel DLA in samples extracted from different organs [4,5]. This contrasts with HDV, which is strictly hepatotropic [25]. However, this hepato-tropism is mediated by the specific interaction of the hepatic NTCP receptor and the HBV envelope.

To investigate DLA replication in tissues other than the liver, we took advantage of three non-hepatic cell lines. HeLa cells were isolated from a cervical carcinoma derived from a 31-year-old patient. A549 was isolated from the lung of a 58-year-old male with carcinoma. HEK293T cell line was derived by transformation of primary cells from human embryonic kidney with adenovirus. Nonetheless, the cell line associates with many neuronal phenotypes. Therefore, the cell line might be of neuronal origin rather than of kidney origin [26].

When investigating replication in non-liver-derived cell lines, the intracellular antigen level indicated comparable expression of HDAg, WoAg, and DeAg, as demonstrated by Western blotting, in HeLa and HEK293T cells (Figure 4B). Nevertheless, CDMS resulted in an increase in clusters number and size for WoDV and DeDV compared to HDV, indicating a stronger spreading capability in non-hepatic tissues. In A549 cells, all the delta agents could not replicate efficiently, implying either a lack of cellular factors required for replication or the presence of potent restriction factors controlling replication. Since HDV cell-division-mediated spread is highly sensitive to IFN-induced genes, such factors may also play a role in controlling CDMS of DLA.

Little is known about if and how DLA could spread extracellularly. Since they lack large delta antigens, packaging by envelope proteins via farnesylation is presumably not supported. Therefore, the successful complementation of RNPs from DLA with L-HDAg and even successful packaging into HBV envelopes not only indicates a functional interaction between L-HDAg and DLA genomes but also that this interaction allows farnesylation of the artificially provided L-HDAg. This efficient packaging of WoDV and DeDV RNPs by HBsAg results in the pseudotyping of HDV-like agents with HBV envelope proteins and infection of hepatocytes in an NTCP-dependent manner. We could speculate that the RNPs of WoDV and DeDV might have the ability to heteromultimerize with the L-HDAg, facilitating the formation of a functional complex. This interaction could enhance the efficiency of the entire assembly, promoting its subsequent packaging by HBsAg. This finding provides an important tool to study replication upon synchronized infection events such as investigation of innate immune stimulation in infected cells and comparative studies. It also implies that adaptation to HBV envelopes occurred only recently during evolution and has not even occurred for WoDV. Remarkably, earlier studies demonstrated that HDV RNPs can adapt to the Woodchuck Hepatitis Virus (WHV) envelope proteins for viral particle formation [27,28].

If we look at our results from an evolutionary point of view, we could speculate that the human HDV gained the capability to express a farnesylated large delta antigen, probably in co-existence with the human HBV in the human liver. This interaction permitted an efficient adaptation of the human HDV as hepatotropic virus. On the other hand, L-HDAg restricts intracellular HDV replication. Maintaining a balance between L-HDAg-mediated HDV release and intracellular replication may be critical for the establishment of a chronic infection in the liver.

## 5. Conclusions

In summary, we investigated the replication, spread, and pseudotyping of mammalian DLA in cells from different hosts and tissues. Our study provides novel insights into the evolution of HDV and DLA in regards of genome diversity, host adaptation, and helper virus selection. It also sheds light on the importance of cell division-mediated spread for HDV persistence, which should be considered for developing a curative treatment.

## Data Availability

Genome sequences of delta agents investigated in this study are available online through the following links: WoDV (MmonDV): (https://raw.githubusercontent.com/wiki/ababaian/serratus/assets/murray.fa) accessed on 15 April 2021; DeDV (OvirDV): (https://raw.githubusercontent.com/wiki/ababaian/serratus/assets/bambi.fa) accessed on 15 April 2021; BaDV (PmacDV): (https://raw.githubusercontent.com/wiki/ababaian/serratus/assets/lassie.fa) accessed on 15 April 2021. For each DLA, run RSA and study RSA codes are provided in Table S1.

## Supplementary Materials

The following supporting information can be downloaded at: https://www.mdpi.com/article/10.3390/v16060859/s1, Table S1. SRA run and SRA study ID of the respective HDV and HDV-like agents genome sequence; Table S2. Plasmids used in this study; Table S3. RT-qPCR primers for viral RNA detection. Table S4. Viruses and pseudo particles used in this study; Table S5. Primary and secondary antibodies used in this study. Figure S1. Alignment of the large delta antigen sequences of the HDV genotypes and putative large delta antigen of selected DLA; Figure S2. Schematic representation of HDV and mammalian delta agents’ genomes used in this study; Figure S3. Northern blot, Western blot, and viral amplification assay of HuH7 cells transfected with HDV replication defect cDNA; Figure S4. Autonomous extracellular spread of delta agents is rare.

## References

[1] Rizzetto M., Canese M.G., Arico S., Crivelli O., Trepo C., Bonino F., Verme G. (1977). Immunofluorescence detection of new antigen-antibody system (delta/anti-delta) associated to hepatitis B virus in liver and in serum of HBsAg carriers. Gut.

[2] Wille M., Netter H.J., Littlejohn M., Yuen L., Shi M., Eden J.S., Klaassen M., Holmes E.C., Hurt A.C. (2018). A Divergent Hepatitis D-Like Agent in Birds. Viruses.

[3] Hetzel U., Szirovicza L., Smura T., Prahauser B., Vapalahti O., Kipar A., Hepojoki J. (2019). Identification of a Novel Deltavirus in Boa Constrictors. mBio.

[4] Chang W.S., Pettersson J.H., Le Lay C., Shi M., Lo N., Wille M., Eden J.S., Holmes E.C. (2019). Novel hepatitis D-like agents in vertebrates and invertebrates. Virus Evol..

[5] Bergner L.M., Orton R.J., Broos A., Tello C., Becker D.J., Carrera J.E., Patel A.H., Biek R., Streicker D.G. (2021). Diversification of mammalian deltaviruses by host shifting. Proc. Natl. Acad. Sci. USA.

[6] Iwamoto M., Shibata Y., Kawasaki J., Kojima S., Li Y.T., Iwami S., Muramatsu M., Wu H.-L., Wada K., Tomonaga K. (2021). Identification of novel avian and mammalian deltaviruses provides new insights into deltavirus evolution. Virus Evol..

[7] Paraskevopoulou S., Pirzer F., Goldmann N., Schmid J., Corman V.M., Gottula L.T., Schroeder S., Rasche A., Muth D., Drexler J.F. (2020). Mammalian deltavirus without hepadnavirus coinfection in the neotropical rodent *Proechimys semispinosus*. Proc. Natl. Acad. Sci. USA.

[8] Giersch K., Bhadra O.D., Volz T., Allweiss L., Riecken K., Fehse B., Lohse A.W., Petersen J., Sureau C., Urban S. (2018). Hepatitis delta virus persists during liver regeneration and is amplified through cell division both in vitro and in vivo. Gut.

[9] Zhang Z., Ni Y., Lempp F.A., Walter L., Mutz P., Bartenschlager R., Urban S. (2022). Hepatitis D virus-induced interferon response and administered interferons control cell division-mediated virus spread. J. Hepatol..

[10] Jayan G.C., Casey J.L. (2002). Increased RNA editing and inhibition of hepatitis delta virus replication by high-level expression of ADAR1 and ADAR2. J. Virol..

[11] Lee C.Z., Chen P.J., Lai M.M., Chen D.S. (1994). Isoprenylation of large hepatitis delta antigen is necessary but not sufficient for hepatitis delta virus assembly. Virology.

[12] Glenn J.S., Watson J.A., Havel C.M., White J.M. (1992). Identification of a prenylation site in delta virus large antigen. Science.

[13] Sureau C., Guerra B., Lanford R.E. (1993). Role of the large hepatitis B virus envelope protein in infectivity of the hepatitis delta virion. J. Virol..

[14] Szirovicza L., Hetzel U., Kipar A., Hepojoki J. (2022). Short ‘1.2x Genome’ Infectious Clone Initiates Kolmiovirid Replication in Boa constrictor Cells. Viruses.

[15] Szirovicza L., Hetzel U., Kipar A., Martinez-Sobrido L., Vapalahti O., Hepojoki J. (2020). Snake Deltavirus Utilizes Envelope Proteins of Different Viruses To Generate Infectious Particles. mBio.

[16] Prasad V., Cerikan B., Stahl Y., Kopp K., Magg V., Acosta-Rivero N., Kim H., Klein K., Funaya C., Haselmann U. (2023). Enhanced SARS-CoV-2 entry via UPR-dependent AMPK-related kinase NUAK2. Mol. Cell.

[17] Wang W., Lempp F.A., Schlund F., Walter L., Decker C.C., Zhang Z., Ni Y., Urban S. (2021). Assembly and infection efficacy of hepatitis B virus surface protein exchanges in 8 hepatitis D virus genotype isolates. J. Hepatol..

[18] Hartwig D., Schutte C., Warnecke J., Dorn I., Hennig H., Kirchner H., Schlenke P. (2006). The large form of ADAR 1 is responsible for enhanced hepatitis delta virus RNA editing in interferon-alpha-stimulated host cells. J. Viral Hepat..

[19] Hwang S.B., Lai M.M. (1994). Isoprenylation masks a conformational epitope and enhances trans-dominant inhibitory function of the large hepatitis delta antigen. J. Virol..

[20] Modahl L.E., Lai M.M. (2000). The large delta antigen of hepatitis delta virus potently inhibits genomic but not antigenomic RNA synthesis: A mechanism enabling initiation of viral replication. J. Virol..

[21] Flores R., Grubb D., Elleuch A., Nohales M.A., Delgado S., Gago S. (2011). Rolling-circle replication of viroids, viroid-like satellite RNAs and hepatitis delta virus: Variations on a theme. RNA Biol..

[22] Netter H.J., Barrios M.H., Littlejohn M., Yuen L.K.W. (2021). Hepatitis Delta Virus (HDV) and Delta-Like Agents: Insights Into Their Origin. Front. Microbiol..

[23] Taylor J., Pelchat M. (2010). Origin of hepatitis delta virus. Future Microbiol..

[24] Khalfi P., Denis Z., McKellar J., Merolla G., Chavey C., Ursic-Bedoya J., Soppa L., Szirovicza L., Hetzel U., Dufourt J. (2024). Comparative analysis of human, rodent and snake deltavirus replication. PLoS Pathog..

[25] Ni Y., Lempp F.A., Mehrle S., Nkongolo S., Kaufman C., Fälth M., Stindt J., Königer C., Nassal M., Kubitz R. (2014). Hepatitis B and D viruses exploit sodium taurocholate co-transporting polypeptide for species-specific entry into hepatocytes. Gastroenterology.

[26] Shaw G., Morse S., Ararat M., Graham F.L. (2002). Preferential transformation of human neuronal cells by human adenoviruses and the origin of HEK 293 cells. FASEB J..

[27] Ryu W.S., Bayer M., Taylor J. (1992). Assembly of hepatitis delta virus particles. J. Virol..

[28] Ponzetto A., Cote P.J., Popper H., Hoyer B.H., London W.T., Ford E.C., Bonino F., Purcell R.H., Gerin J.L. (1984). Transmission of the hepatitis B virus-associated delta agent to the eastern woodchuck. Proc. Natl. Acad. Sci. USA.

