# Supplementary material for: Analysis of Replication, Cell Division-Mediated Spread, and HBV Envelope Protein-Dependent Pseudotyping of Three Mammalian Delta-like Agents
Source: Viruses. 2024 May 28;16(6):859. doi: 10.3390/v16060859 (PMC11209313; doi:10.3390/v16060859)
Supplement: Supplementary file 1 [file viruses-16-00859-s001.zip › viruses-3012235-supplementary.pdf]

Supplementary Information for

Analysis of replication, cell division-mediated spread, and HBV envelope protein-dependent pseudotyping of three mammalian delta-like agents.

Gnimah Eva Gnouamozi [1], Zhenfeng Zhang [1,2], Vibhu Prasad [1], Chris Lauber [3,4,5], Stefan Seitz [6,7], Stephan Urban [1,6]

1 Department of Infectious Diseases, Molecular Virology, University Hospital Heidelberg, Heidelberg, Germany
2 School of Public Health and Emergency Management, Southern University of Science and Technology, Shenzhen, China
3 Institute for Experimental Virology, TWINCORE Centre for Experimental and Clinical Infection Research, a joint venture between the Hannover Medical School (MHH) and the Helmholtz Centre for Infection Research (HZI), Hannover, Germany
4 German Center for Infection Research (DZIF), Hannover Partner Site, Hannover, Germany
5 Cluster of Excellence 2155 RESIST, Hannover, Germany
6 German Center for Infection Research (DZIF), Heidelberg Partner Site, Heidelberg, Germany
7 German Cancer Research Center (DKFZ), Division of Virus-Associated Carcinogenesis, Heidelberg, Germany

Supplementary materials and methods

Cell lines and cell culture. HuH7 hepatocarcinoma cells and HuH7-NTCP cells (subclone derived from HuH7 cells by lentiviral overexpression of NTCP), HEK293T kidney cells, A549 lung adenocarcinoma cells, HeLa uterus adenocarcinoma cells and VeroE6 African green monkey kidney cells were maintained in Dulbecco's Modified Eagle Medium (DMEM), supplemented with 10% FCS, 2 mM L-glutamine, 50 U/mL penicillin, 50 µg/mL streptomycin. CHO - Chinese hamster ovary cells, PaKi - Bat kidney cells and LMH – Chicken hepatoma cells were kept in culture with F12 MEM medium supplemented with 10% of FCS, 2 mM L-glutamine, 50 U/mL penicillin, 50 µg/mL streptomycin, Pyruvate and NEAA. PaKi cells were a kind gift from Martin Schwemmle, University of Freiburg; A549 cells were kindly provided by Ralf Bartenschlager – Heidelberg University.

Plasmids. pJC126, a pcDNA-based eukaryotic expression vector encoding an overlength antigenome of HDV Gt-1 was provided by Dr. John Taylor. pcDNAHDVgt5, a eukaryotic expression vector encoding a replication-defective overlength antigenome of HDV Gt-5 clone was described before (Wang et al., 2021). pT7HB2.7plasmid encoding a 2.7 kb subgenomic fragment of HBV Gt-B including the L, M, S & X ORFs under the authentic

promoters. For the novel mammalian delta viruses cDNAs encoding viral sequences (Table S1) were synthesized and cloned into pcDNA vectors. The final plasmids encode a 1.1-fold overlength antigenomic sequence of the respective HDV-like genomes under a CMV promoter. Plasmid details are listed in Table S2.

Infection. For infection experiments, HuH7-NTCP cells were seeded in 24-well plates (1.5x10^5/well). The following day infection medium was prepared containing PEG precipitated virus, DMSO (final concentration 2%) and PEG8000 (4% final concentration). After 16 hours cells were washed twice with PBS, and medium (complete DMEM, 2% DMSO) was changed at day 1 and 3 post infection (p.i.). As an entry inhibition control, the cells were treated with 500 nM Bulevirtide during infection. At day 7 p.i. cells were fixed for immunofluorescence (IF) experiment.

RNA purification from precipitated virus and RT-qPCR detection. To determine the concentration of the virus in the concentrated supernatant the QiAMP Viral RNA Mini kit was used, following the manufacturer's instructions. Total viral RNA was extracted from 120 µL virus. To remove residual plasmid, 40 µL of the eluate was digested with DNase I (2 U/µL) at 37°C for 15 min. To inactivate DNase and to break the secondary structure of HDV RNA, the RNA was incubated for 5 minutes at heat shock at 95°C. Reverse transcription was performed according to the High-capacity cDNA reverse transcription kit using 10 µL of extracted and DNAse treated RNA. The cDNA was diluted 1:5 with Braun water and RT- qPCR with SYBR green was performed in duplicate wells using primer delisted in Table S3.

RNA purification and RT-qPCR of intracellular RNA. RNA extraction from samples collected at day 2, 6 or 12 post transfection (p.t) was carried out using the NucleoSpin RNA Kit following the manufacturer's instructions. RNA concentration was measured by Nanodrop, and 1 µg of total RNA was reverse transcribed as described above. RT- qPCR with SYBR green was performed in duplicate wells using primer delisted in Table S3.

Western blot and antibodies. For western blot analysis, the cells grown in 24 well plates were washed once with PBS, then lysed in 75 µL 2x SDS sample buffer. The lysates were stored at -20°C. Before loading the SDS gel, the samples were vortexed, heated to 95°C for 10 min, and the liquid was centrifuged at maximum speed for 20 minutes. For SDS

PAGE, a resolving gel containing 15% polyacrylamide was combined with a 3% stacking gel. For each well, 3 µl of lysate was added. After resolving in SDS running buffer at 90 V for 20 min then at 120 V, the proteins were transferred from the gel to a nitrocellulose membrane using a semi-dry system at 25 V for 30 min. Directly after blotting, the membrane was incubated in blocking buffer for 1 hour at RT. The membrane was incubated with primary antibody solution: FD3A7 antibody for delta antigen detection (1:3,000 dilution in blocking buffer) and mouse anti actin (Sigma Aldrich, cat.no. A5441, clone no. AC15 ,1:5,000 dilution in blocking buffer) at 4°C, ON. After washing thrice with TBST for 10 min, secondary antibodies (goat anti rabbit Alexa Fluor 800, and goat anti mouse Alexa Fluor 680 both 1:10,000 in blocking buffer) was applied for 1 h at RT. After additional 2 TBST and 1 last PBS washes, acquisition was performed using LI-COR Odyssey Imaging Systems.

Immunofluorescence and antibodies. Cells were washed once with PBS, then fixed with 4% PFA at RT for 30 min. After three washing steps with PBS, permeabilization buffer was added, the cells were permeabilized at RT for 30 min and washed thrice with PBS. The cells were incubated with FD3A7 antibody diluted 1:3,000 in 2% BSA/PBS at RT for 1 hour. After three washing steps with PBS, cells were incubated with 1:1000 diluted secondary antibody (goat anti rabbit Alexa Fluor 546, Invitrogen) and 2 µg/mL Hoechst 33342 stain in the same solution as the secondary antibody for 1 hour at RT while shaking, protected from light. Until imaging, the stained cells were stored in the dark at 4°C. Cells seeded on coverslips were stained as described above. Then, the coverslips were once washed in de-ionized H2O and mounted on glass slides with 10 µL of Fluoromount-G mounting medium. The cells were imaged at inverted microscopes. For sub-cellular localization, images were taken at 20x magnification.

Northern Blot. RNA extraction was performed as mentioned above (see section: **RNA purification and RT-qPCR of intracellular RNA)**. 10 µg of RNA was run in a 1.5% MOPS agarose gel containing 2.2M formaldehyde. After denaturation (50mM NaOH for 5min), RNAs were transferred to a nylon membrane by capillary transfer using 20× SSC buffer. Membranes were dried and fixed by UV crosslinking. Virus specific probes were synthetized via in vitro transcription using the DIG RNA labeling Mix, 10 x conc protocol

(cat.No. 11277073910). Membranes were hybridized at 60 °C overnight singularly and visualized using a luminescent DIG detection kit (Roche).

Genome sequences

Viral agent	SRA_run	SRA_study
HDV	M21012	
WoDV	SRR2136906	SRP061879
DeDV	SRR4256033	SRP083076
BaDV	SRR7910143	SRP162769

Table S1.SRA run and SRA study ID of the respective HDV and HDV-like agents genome sequences**.**

Plasmids

Plasmid name	Description	Reference
pJC126	Eukaryotic expression vector encoding an overlength (1.1) antigenome of Hepatitis Delta Virus genotype 1	Gudima et al., (2002)
pcDNA3.1/Zeo (+) WoDV 1.1(-)	Eukaryotic expression vector encoding an overlength (1.1) antigenome of woodchuck delta agent	This study
pcDNA3.1/Zeo (+) DeDV 1.1(-)	Eukaryotic expression vector encoding an overlength (1.1) antigenome of deer delta agent	This study
pcDNA3.1/Zeo(+) BaDV 1.1(-)	Eukaryotic expression vector encoding an overlength (1.1) antigenome of bat delta agent	This study
pcDNA3.1/Zeo(+)-HDVgt5Senegal (defect)	Eukaryotic expression vector encoding an overlength (1.1) antigenome of Hepatitis Delta Virus genotype 5, replication defective	Wang et al., (2021)
pcDNA3.1/Zeo(+)-L-HDAg	Eukaryotic expression vector encoding the large HDAg with the editing mutation	This study
pT7HB2.7	Plasmid encoding a 2700 bp subgenomic fragment of HBV including the L, M, S & X ORFs under the authentic promoter	Sureau et al., (2003)

Table S2. Plasmids used in this study**.**

Primers

Name	Sequence (5' to 3')	Source
HDV-Ferns_for	GCGCCGGCYGGGCAAC	Ferns et al., (2012)
HDV-Ferns_rev	TTCCTCTTCGGGTCGGCATG	
HDV_for	ATGAGCCGGTCCGAGTCGAGGAAGA	This study
HDV_rev	TTCTTTCTTCCGGCCACCCACTGC	
WoDV_for	CCTGGCTGGGGAACATCCTGGGAAT	This study
WoDV_rev	TTCTCCTCGTGGTCTCTTGGACGGG	
DeDV_for	AATCCCTGGCTGGGAAACGTCCTCG	This study
DeDV_rev	ATCCGATCTTGGTCTCTTGGCCGGG	
BaDV_for	AACCCATGGCTGGGGAACGTTCTTG	This study
BaDV_rev	GCGTCTTTTCTTAGCCTGGGGAGCG	

Table S3. RT-qPCR primers for viral RNA detection.

Viruses and pseudoparticles

**Virus**	**Viral (pseudo) particle for infection assay**	**Source**
HDV	Cell-culture derived, produced in HuH7 cells by co-transfection and purified via PEG precipitation or heparin affinity chromatography.	This study
WoDV/ HBsAg	Cell-culture derived, produced in HuH7 cells by co-transfection and L-HDAg complementation and purified via PEG precipitation or heparin affinity chromatography.	This study
DeDV/ HBsAg	Cell-culture derived, produced in HuH7 cells by co-transfection and L-HDAg complementation and purified via PEG precipitation or heparin affinity chromatography.	This study

Table S4. Viruses and pseudo particles used in this study**.**

Primary and secondary antibodies

Name	Dilution	Reference	Supplier	Cat. No.	Clone no.
HDAg	IF 1:3000<br>WB 1:3000	Wang et al., (2021)	Kerafast	EHD001	FD3A7
β-actin	WB 1:5000	Zhang et al., (2018)	Sigma-Aldrich	A5441	AC15

Name	Dilution	Supplier	Cat. No.
Goat anti-rabbit AlexaFluor 488	IF 1:1000	Thermo Fisher Scientific	A11008
Goat anti-rabbit AlexaFluor 546	IF 1:1000	Thermo Fisher Scientific	A11010
Goat anti-rabbit AlexaFluor 647	IF 1:1000	Thermo Fisher Scientific	A21244
Goat anti-rabbit IRDye 800CW	WB: 1:10000	Licor	
Goat anti-mouse IRDye 680CW	WB: 1:10000	Thermo Fisher Scientific	A21057

Table S5. Primary and secondary antibodies used in this study.

Supplementary information

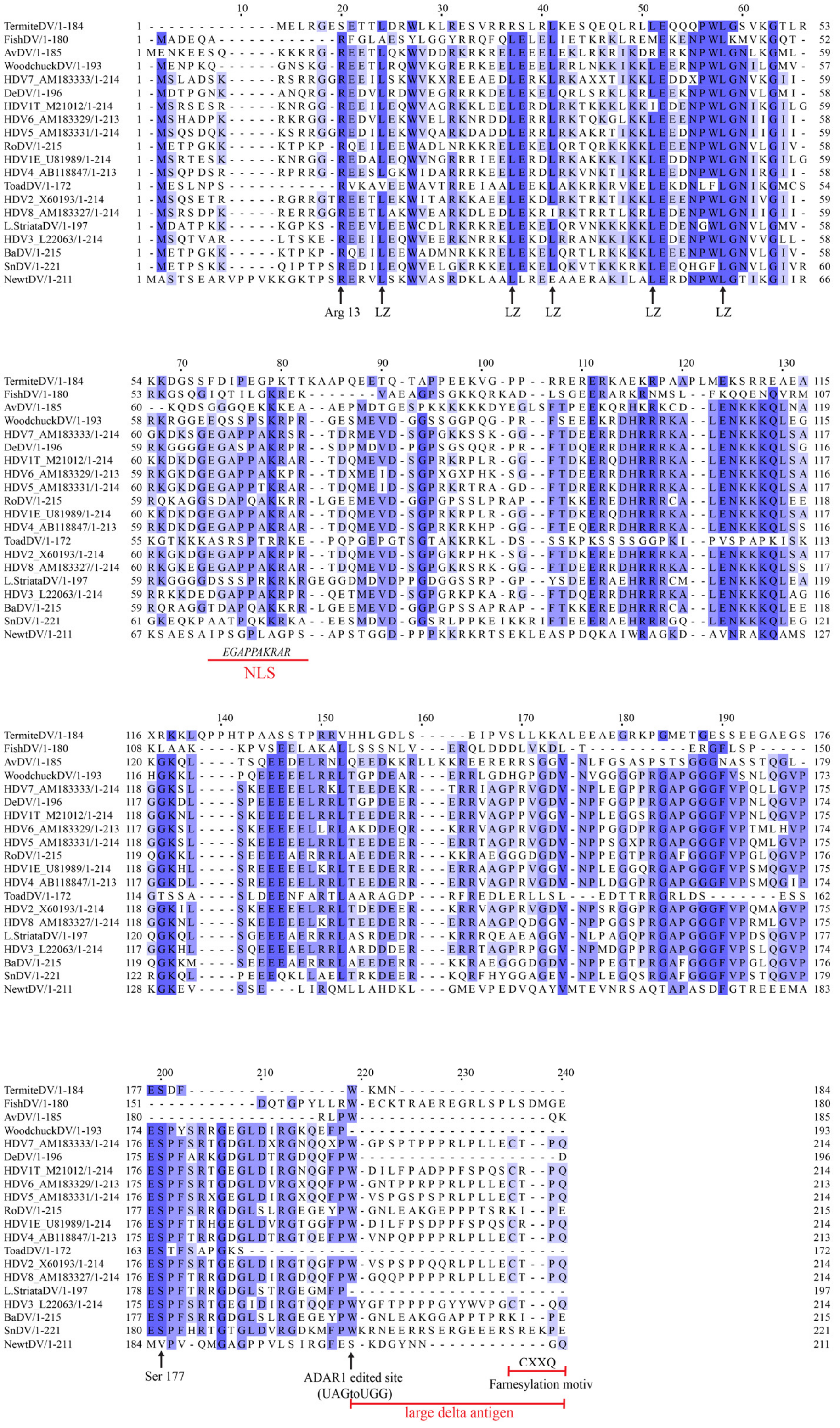

FigureS1. Alignment of the large delta antigen sequences of the HDV genotypes and putative large delta antigen of selected DLA. The translated genome of human HDV is compared with the putative L-DAg of newly discovered delta agents. The translations of the L-DAg proteins were aligned using MUSCLE. The conserved regions sharing similar

signatures between different DAgs are marked in blues. The consensus sequence is obtained considering a threshold of ≥ 50% of amino acid identity.

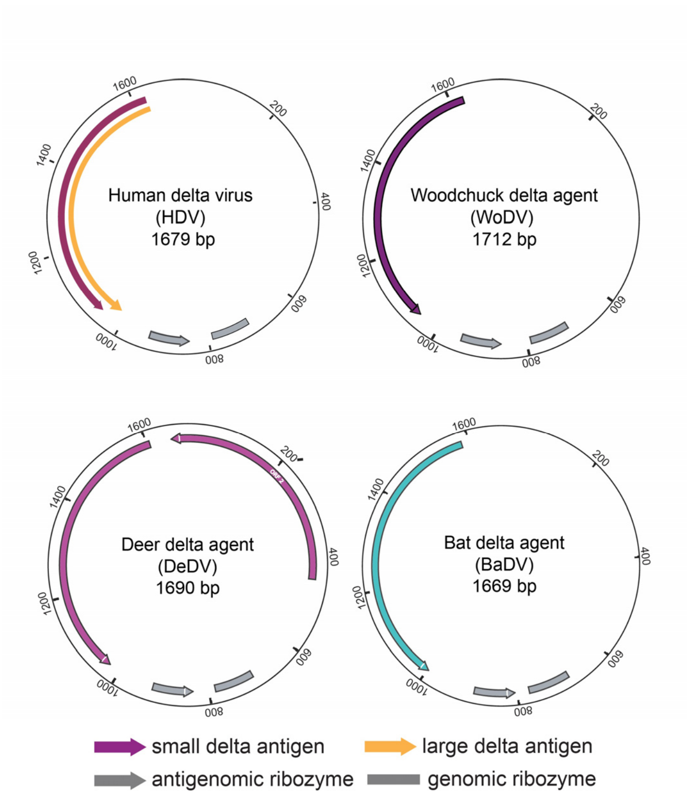

FigureS2. Schematic representation of HDV and mammalian delta agents' genomes used in this study.

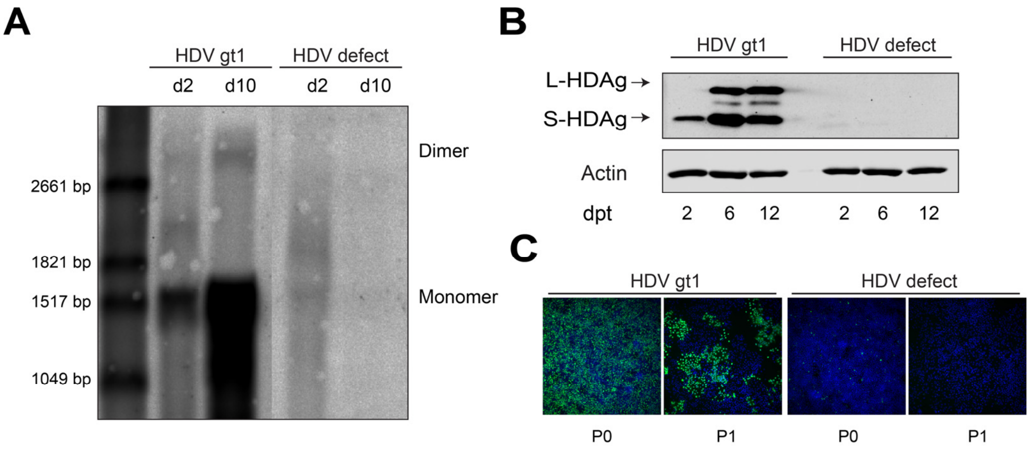

FigureS3. Northern blot, western blot, and viral amplification assay of HuH7 cells transfected with HDV replication defect cDNA. HuH7 cells were transfected with pcDNA3.1-HDV-defect (replication defect control). RNA was analyzed by northern blot at day 2 and 10 post transfection (A) and HDAg was detected via western at day 2, 6 and 12 post transfections (B). As negative control, HuH7 were also transfected with pcDNA3.1-HDV-defect and split as perform in *Fig. 2*.

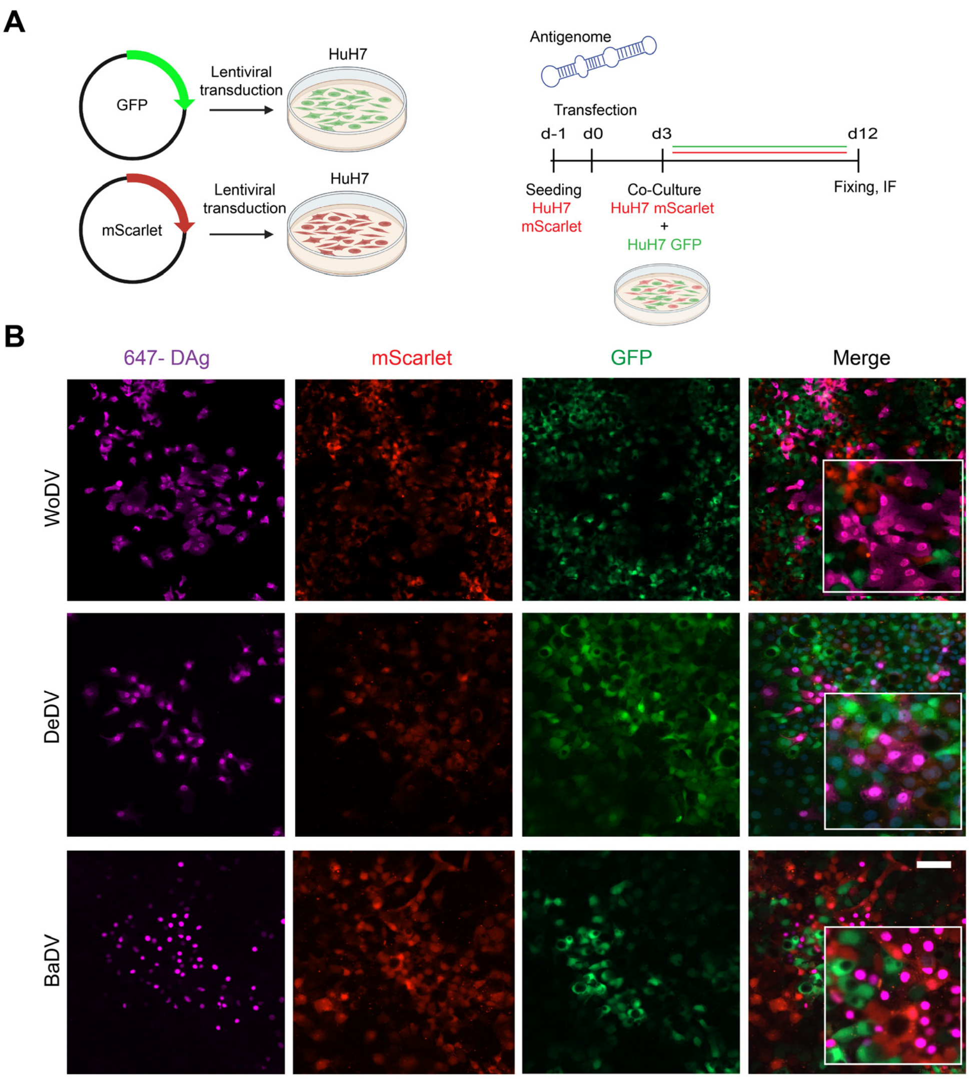

FigureS4. Autonomous extracellular spread of delta viruses is rare. HuH7 were transduced for the expression of mScarlet or GFP proteins and maintained under antibiotic selection for several passages. HuH7 mScarlet cells were transfected with delta-like agents constructs and after 3 days they were co-cultured with HuH7 GFP cells in presence of DMSO (2%). After 12 days cells were fixed and stained for delta antigen (magenta) visualization (FD3A7 anti-S-HDAg and Alexa Fluor 647-labeled anti-rabbit antibody). Images were acquired using ZEISS Cell discoverer 7. (Scale: 50 µm)

Wang, W., Lempp, F. A., Schlund, F., Walter, L., Decker, C. C., Zhang, Z., Ni, Y., & Urban, S. (2021). Assembly and infection efficacy of hepatitis B virus surface protein exchanges in 8 hepatitis D virus genotype isolates. *J Hepatol*, *75*(2), 311-323. https://doi.org/10.1016/j.jhep.2021.03.025
